# High glucose enhances LPS-stimulated human PMVEC hyperpermeability via the NO pathway

**DOI:** 10.3892/etm.2013.1166

**Published:** 2013-06-18

**Authors:** XIU-JUAN LIU, ZHI-DAN ZHANG, XIAO-CHUN MA

**Affiliations:** Department of Intensive Care Unit, The First Affiliated Hospital, China Medical University, Shenyang, Liaoning 110001, P.R. China

**Keywords:** high glucose, nitric oxide synthase, dimethylarginine dimethylaminohydrolase, endothelial permeability

## Abstract

Chronic hyperglycemia is an established risk factor for endothelial damage. It remains unclear, however, whether brief hyperglycemic exposure exacerbates the damage to vascular endothelial cells induced by endotoxin. We hypothesize that brief hyperglycemic exposure enhances the permeability of the endothelium following stimulation with lipopolysaccharide (LPS). Correlations between modulation of nitric oxide synthase (NOS) pathways and altered endothelial homeostasis have been studied and demonstrated in various pathophysiological conditions. NOS activities are regulated by endogenous inhibitors, including asymmetric dimethylarginine (ADMA), which is metabolized by dimethylarginine dimethylaminohydrolase (DDAH). Since previous data demonstrated that endothelial dysfunction may be related to reduced expression and/or activity of DDAH, in this study, we aimed to determine the effect of increased glucose levels on pulmonary microvascular endothelial cell (PMVEC) permeability, including effects on the NOS pathways. Human PMVECs were incubated with normal (5.5 mM) and high (33 mM) concentrations of D-glucose for 5 days to create a monolayer of cells prior to LPS stimulation (10 *μ*g/ml) for 12 h. When stimulated with LPS, cells incubated with a high glucose (HG) concentration had significant microfilament rearrangement compared with cells incubated with a normal glucose concentration, as determined by immunofluorescence. Scanning electron microscopy revealed a larger average diameter and increased number of fenestrae on the hyperglycemic PMVECs when stimulated with LPS, compared with PMVECs cultured with a normal glucose concentration. The results demonstrated that a high concentration of glucose increases the LPS-stimulated horseradish peroxidase (HRP) permeability compared with a normal concentration of glucose. Furthermore, a HG concentration upregulated LPS-stimulated inducible NOS (iNOS) production and down-regulated endothelial NOS (eNOS) and DDAH-2 expression. Hyperglycemia significantly increased LPS-stimulated nitrite/nitrate production (stable NO end-products). Our results, thus, demonstrate that *in vitro* HG concentrations exacerbate LPS-stimulated cytoskeletal rearrangement and hyperpermeability of an endothelial monolayer, and cause further imbalance of the NO pathway. These results suggest that it is important to manage even short-term increases in blood glucose, particularly following acute infection.

## Introduction

There is increasing evidence that the endothelium plays a central and pathogenic role in sepsis. When exposed to certain agonists, including lipopolysaccharide (LPS), endothelial cells become activated. The activation state is manifested by enhanced permeability, increased leukocyte adhesion, a shift in the hemostatic balance towards a procoagulant phenotype and altered regulation of vasomotor tone (1). Hyperglycemia, be it secondary to diabetes, impaired glucose tolerance or impaired fasting glucose, or stress-induced, is common in individuals with critical illnesses, including sepsis. Thus, the concurrent existence of hyperglycemia and endotoxemia is common. Chronic hyperglycemia (diabetes mellitus) is itself known to activate the endothelium and individuals with this condition have a higher rate of mortality from sepsis compared with their non-diabetic septic counterparts. Chronic diabetes causes endothelial systems to have higher sensitivity to septicemia (2–3). For instance, diabetes is associated with exacerbated LPS-induced immune and hemostatic responses. The increased glucose concentration in diabetes mellitus is associated with enhanced LPS-stimulated transcriptional activator protein (AP)-1 and nuclear factor-κB (NF-κB) activity, which are important in the transcriptional activation of genes involved in inflammation (4). In addition, the exposure of mononuclear cells to high glucose (HG) augments the LPS-stimulated expression of matrix metalloproteinase (MMP) (5) and pro-inflammatory cytokines, including tumor necrosis factor (TNF)-α and interleukin (IL)-8 (6–8). HG and LPS levels also increase the generation of reactive oxygen species by peritoneal macrophages (9). Stegenga *et al* demonstrated that hyperglycemia enhances coagulation whereas hyperinsulinemia inhibits fibrinolysis during human endotoxemia (10). It remains unclear, however, whether brief hyperglycemic episodes alter the function of vascular endothelial cells in response to endotoxins. We hypothesize that brief hyperglycemic episodes enhance the permeability of microvascular endothelial cells induced by LPS *in vitro.*

The endothelium constitutes the inner lining of blood vessels and regulates the exchange of fluids, macromolecules and leukocytes between blood and interstitial tissues. Precise control of the endothelial barrier function strongly depends on endothelial nitric oxide (NO) production, since inhibition of NO production and excessive amounts of NO induce vascular leakage (11). Basal NO levels are necessary for vasodilation, platelet aggregation and the modulation of inflammatory cell adhesion to the endothelium (12–14). The effects of NO on the cardiovascular system depend on the amount of NO produced, the local environment and the redox state of NO. While low NO levels are necessary for endothelial integrity, excessive NO is pathogenic, compromising barrier function (15). NO is produced by three different NO synthase (NOS) isoforms: neuronal (nNOS), endothelial (eNOS) and inducible NOS (iNOS). NOS activity is regulated by endogenous inhibitors, including asymmetric dimethylarginine (ADMA), which is metabolized by dimethylarginine dimethylaminohydrolase (DDAH). Two distinct DDAH isoforms have been described; DDAH-1 is typically located in tissues expressing nNOS, whereas DDAH-2 predominates in tissues containing eNOS (16). Since previous data demonstrated that endothelial dysfunction may be related to reduced activity of DDAH (17), we hypothesize that there is a dynamic balance between the protective and pathogenic roles of NO. This balance may be regulated by the location, time and magnitude of NO release. We also hypothesize that the DDAH/eNOS/iNOS stress reaction to insults of brief hyperglycemic episodes and LPS is aggravated. The current study was conducted with the aim of investigating these hypotheses.

## Materials and methods

### Cell culture

Human pulmonary microvascular endothelial cells (PMVECs) were purchased from American Type Culture Collection (ATCC; Manassas, VA, USA) and grown in Dulbecco’s modified Eagle’s medium (DMEM) with 10% fetal bovine serum (Gibco-BRL, Invitrogen, Carlsbad, CA, USA). Cells were incubated at 37°C in a 5% CO_2_ humidified atmosphere and maintained at subconfluency by passaging with trypsin-ethylenediaminetetraacetic acid (EDTA; Gibco-BRL). Cells were incubated with normal (5.5 mM) or high (33 mM) D-glucose concentrations (Sigma, St. Louis, MO, USA) for 5 days in medium with 2% serum (to maintain the cells in the quiescent state) and then incubated with LPS (0.00, 0.01, 0.10, 1.00, 10 or 100 *μ*g/ml) for 0, 8, 12, 24 or 36 h. To evaluate the potential cytotoxicity of these agents, MTT assays were performed to assess cell viability. Briefly, cells were plated in 96-well plates (0.4×10^5^ cells/well) and treated with the agent. The supernatant was then replaced by fresh medium containing 10% MTT. The formazan product was dissolved in dimethyl sulfoxide (DMSO) and the absorbance at 592 nm was measured.

### F-actin staining

Cells were washed with phosphate-buffered saline (PBS) to remove cell debris and fixed in 4% paraformaldehyde (v/v) for 10 min. Following fixation, the cells were permeabilized with 0.5% Triton X-100 (v/v) in PBS and stained with phalloidin-fluorescein isothiocyanate (FITC; Sigma) to label actin and 4′,6-diamidino-2-phenylindole (DAPI) to label cell nuclei in PBS for 45 min at room temperature (22–24°C). Following the final rinse, the cells were mounted on a glass slide with fluorescence mounting medium (Invitrogen Life Technologies, Carlsbad, CA, USA). Stained cells were photographed with an Olympus Provis fluorescence microscope (Olympus, Hamburg, Germany). The images shown are representative of at least three separate experiments.

### Scanning electron microscopy (SEM)

Human PMVEC layers grown on fibronectin-coated dishes (Corning Life Sciences, Corning, CA, USA) were washed with PBS at room temperature. The cells were prefixed with 2.5% glutaraldehyde and post-fixed with 1% osmium tetroxide in 0.05 M PBS for 1 h at 48°C. The cells were then dehydrated in a graded ethanol series and dried using the 100% t-butyl alcohol and finally dried in a JFC-310 freeze drying device (JEOL, Tokyo, Japan). After being coated with gold in a JFC-1100 ion sputter coater (JEOL), samples were examined under a JSM-T300 SEM (JEOL).

### Permeability assay

Human PMVECs were cultivated on polycarbonate membrane Transwell inserts (6.5 mm diameter, 0.4 *μ*m pore size; Corning Life Sciences) coated with ProNectin F until confluent. The amount of culture medium in the upper and lower compartments of the Transwell were 100 and 600 *μ*l, respectively. Prior to the experiment, the culture medium in the two compartments was replaced with fresh medium and 0.126 mM horseradish peroxidase (HRP; type VI-A, molecular weight 44,000; Sigma-Aldrich) was added to the upper compartment. The cells were then incubated under normal culture conditons. After 1 h, the medium in the lower compartment was collected and stored on ice until the HRP enzymatic activity was assayed. Briefly, 60 *μ*l medium was incubated with 860 *μ*l reaction buffer (50 nM NaH_2_PO_4_ with 5 nM guaiacol) for 25 min at room temperature. The reaction was initiated by adding 100 *μ*l hydrogen peroxide. HRP activity was calculated from the increase in absorbance at 470 nm.

### Nitrite/nitrate (NO_2_^−^/NO_3_^−^) quantification

NO production was measured by determining the concentration of nitrite (a stable metabolite of NO) using the Griess reagent system from Jiancheng Institute of Biotechnology (Nanjing, China) according to manufacturer’s instructions. Cell culture medium aliquots (100 *μ*l) were incubated for 30 min at room temperature with 50 mM nicotinamide adenine dinucleotide phosphate (NADPH) and 24 mU nitrate reductase; then, the samples were treated with 0.2 U lactate dehydrogenase and 0.5 mmol sodium pyruvate for 10 min. The color was developed by adding the Griess reagent (1:1, v/v). Finally, after 10 min at room temperature, the absorbance was recorded on a 96-well plate Multiskan Microtiter Plate Reader (Thermo Labsystems, Philadelphia, PA, USA) at 540 nm. Nitrite levels were determined using a standard curve.

### Western blotting

Cells were washed with ice-cold PBS and lysed in RIPA buffer [50 mM Tris (pH 7.5), 150 mM NaCl, 1% NP-40, 0.5% sodium deoxycholate and 0.1% sodium dodecyl sulfate (SDS)] on ice for 30 min. The total protein concentration was determined using a Protein Assay kit (Bio-Rad, Hercules, CA, USA). Protein samples (30–80 *μ*g) were loaded on a 12% SDS-polyacrylamide gel and separated by electrophoresis prior to transfer to polyvinylidene difluoride membranes. After blocking with Tris-buffered saline with Tween-20 [TBST; 20 mM Tris (pH 7.5), 150 mM NaCl and 0.01% Tween-20] containing 5% non-fat dry milk for 1 h at room temperature, the membranes were then incubated with polyclonal anti-DDAH-2 (Santa Cruz Biotechnology, Santa Cruz, CA, USA), monoclonal anti-eNOS (Sigma) and anti-iNOS (Santa Cruz Biotechnology) antibodies overnight at room temperature with constant agitation. The filters were then washed and probed with secondary HRP-linked goat anti-mouse or anti-rabbit antibodies (1:500; Santa Cruz Biotechnology) at room temperature for 1 h. The proteins were detected using an enhanced chemiluminescence (ECL) detection system. Western blots were quantified by densitometric analysis followed by normalization with actin. Results are expressed as arbitrary units (AU).

### Statistical analysis

Data are presented as mean ± standard deviation (SD). One-way analysis of variance and Student’s t-tests were performed to determine the statistically significant differences among different experimental groups. P<0.05 was considered to indicate a statistically significant difference.

## Results

### Cell viability assay

**Figure 1.**
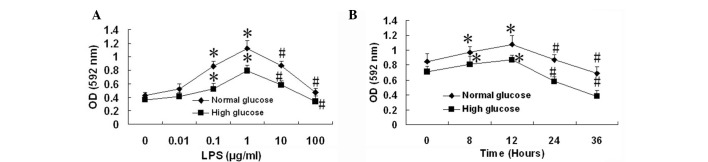
Effect of lipopolysaccharide (LPS) on the cell viability of human pulmonary microvascular endothelial cells (PMVECs). Following the incubation of human PMVECs with normal (5.5 mM) and high (33 mM) D-glucose concentrations for 5 days, the PMVECs were incubated with (A) LPS (0, 0.01, 0.1, 1, 10 and 100 *μ*g/ml) for 12 h or (B) with LPS (10 *μ*g/ml) for 0, 12, 24 and 36 h, respectively. Data are expressed as mean ± standard deviation (SD; n=5). ^*^P<0.05, compared with the LPS (0 *μ*g/ml) group or 0 h group. ^#^P<0.05, compared with the LPS (1 *μ*g/ml) group or 12 h group. OD, optical density.

The MTT assay indicated that the viability of PMVECs was significantly increased by treatment with LPS (0.1 and 1 *μ*g/ml) for 12 h compared with the untreated control (LPS, 0 *μ*g/ml; all P<0.05); however, the viability of the PMVECs was significantly reduced by treatment with higher concentrations of LPS (10 and 100 *μ*g/ml) for 12 h, compared with that of the PMVECs treated with 1 *μ*g/ml LPS in the normal glucose (NG) and HG groups (Fig. 1A; all P<0.05). Then, 10 *μ*g/ml LPS was selected to treat PMVECs for different incubation times. The results indicated that the viability of PMVECs was significantly increased by treatment with LPS (10 *μ*g/ml) for 8 and 12 h compared with the viability prior to treatment with LPS (all P<0.05). However, the viability of the PMVECs was significantly reduced by treatment with LPS (10 *μ*g/ml) for 24 and 36 h compared with the viability following a 12-h treatment with LPS (Fig. 1B; all P<0.05). We therefore used LPS (10 *μ*g/ml) to stimulate cells for 24 h in subsequent experiments.

### Changes of the actin cytoskeleton in PMVECs

**Figure 2.**
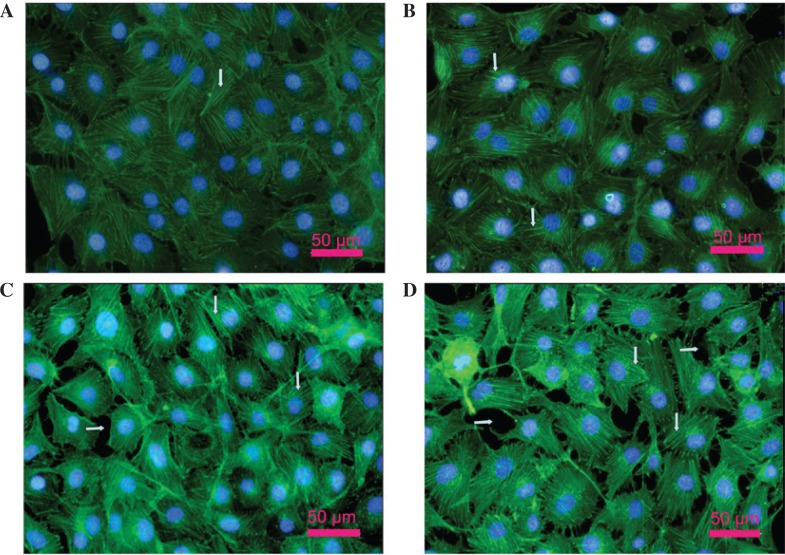
Effect of lipopolysaccharide (LPS) and high glucose on the actin cytoskeleton in human pulmonary microvascular endothelial cells (PMVECs). Cells were incubated with normal (5.5 mM) or high (33 mM) D-glucose concentrations for 5 days in medium with 2% serum (to maintain the cells in the quiescent state) and then cells were incubated with LPS (10 *μ*g/ml) for 12 h. Cells were stained for F-actin and visualized under a fluorescence microscope as described in Materials and methods. Scale bars, 50 *μ*m. (A) Normal glucose group; (B) high glucose group; (C) normal glucose + LPS group; (D) high glucose + LPS group.

As shown in Fig. 2A, the actin cytoskeleton of PMVECs consisted of regular fiber distribution in the cytoplasm and almost continuous peripheral actin fibers at the cell-cell junctions in the NG group. Stress fiber formation was observed in the HG group (vertical arrows in Fig. 2B). In the presence of LPS, cells incubated in NG concentrations had an activated cell phenotype, with thin or no cortical actin and abundant stress fibers (vertical arrows in Fig. 2C). By contrast, when cells incubated in HG concentrations were exposed to LPS, a marked effect on F-actin filament organization was observed, with evident and robust stress fiber formation and intracellular gap formation (parallel arrows in Fig. 2D).

### Formation of fenestrae in PMVECs

**Figure 3.**
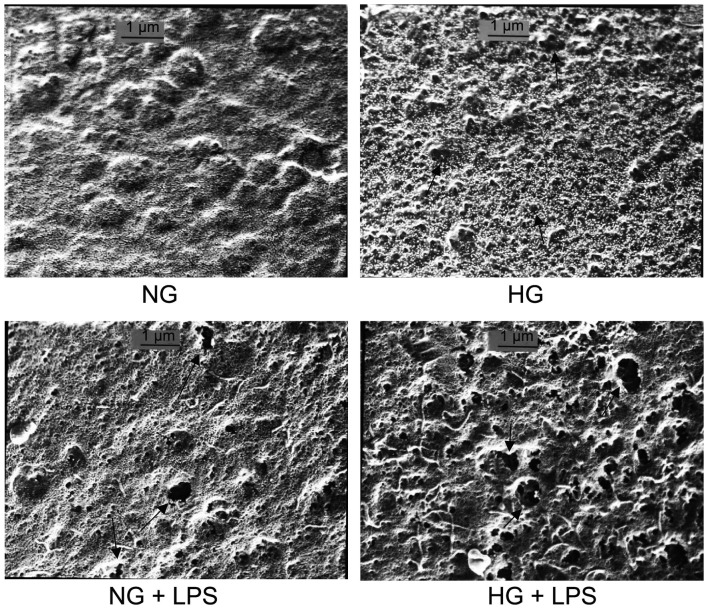
Effect of lipopolysaccharide (LPS) and a high glucose concentration on the number and size of fenestrae in human pulmonary microvascular endothelial cells (PMVECs). Cells were incubated with normal (5.5 mM) or high (33 mM) D-glucose concentrations for 5 days and then incubated with 10 *μ*g/ml LPS for 12 h. Cells were prepared for scanning electron microscopy as described in the Materials and methods. NG, normal glucose; HG, high glucose; NG + LPS, normal glucose + LPS; HG + LPS, high glucose + LPS. Scale bar, 1 *μ*m.

To examine the effect of HG concentrations and LPS on the formation of fenestrae in human PMVECs, cells were incubated with normal (5.5 mM) or high (33 mM) D-glucose concentrations for 5 days, then cells were incubated with 10 *μ*g/ml LPS for 12 h. The samples were prepared for SEM. Results demonstrated that the number and average diameter of fenestrae were significantly increased in the HG and NG + LPS groups compared with those in the NG group (both P<0.05). Compared with the HG or NG + LPS groups, the number and average diameter of fenestrae were significantly increased in the HG + LPS group (both P<0.05; Fig. 3 and Table I).

### Effect of LPS and HG on NO production in human PMVECs

**Figure 4.**
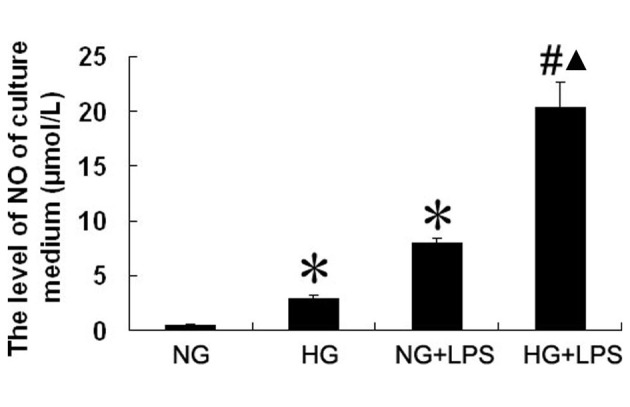
Effect of lipopolysaccharide (LPS) and a high glucose concentration on nitric oxide (NO) production in human pulmonary microvascular endothelial cells (PMVECs). Cells were incubated with normal (5.5 mM) or high (33 mM) D-glucose concentrations for 5 days and then with 10 *μ*g/ml LPS for 12 h. Cell culture medium was collected and the level of NO was measured by chemical methods. NG, normal glucose; HG, high glucose; NG + LPS, normal glucose + LPS; HG + LPS, high glucose + LPS. Data are expressed as mean ± standard deviation (SD; n=5). ^*^P<0.05, compared with the NG group; ^#^P<0.05, compared with the HG group; ^▴^P<0.05, compared with the NG + LPS group.

As shown in Fig. 4, LPS (10 *μ*g/ml) increased the level of NO produced by PMVECs in NG conditions by ∼15-fold compared with the level in the NG group (7.99±0.33 vs. 0.54±0.12 *μ*mol/l, respectively; P<0.05). Compared with the NG group, NO production by PMVECs was significantly increased in the HG group (0.54±0.12 vs. 2.91±0.30 mol/l, respectively; P<0.05). The NO production level of PMVECs was significantly higher in the HG + LPS group than in the NG + LPS group (20.36±2.25 vs. 7.99±0.33 *μ*mol/l, respectively; P<0.05).

### Effect of LPS and HG on the permeability of human PMVECs

**Figure 5.**
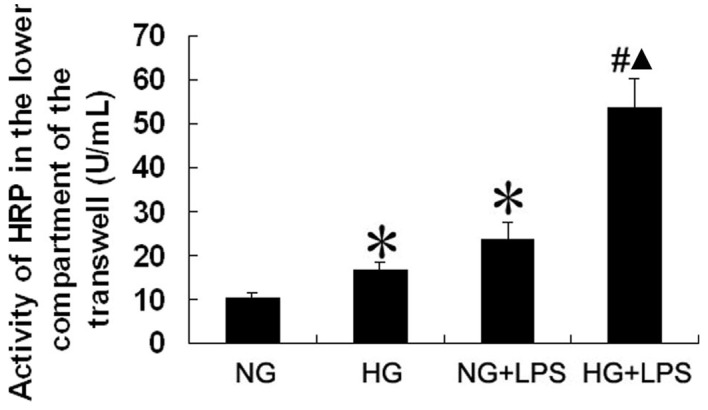
Effect of lipopolysaccharide (LPS) and a high glucose concentration on permeability in human pulmonary microvascular endothelial cells (PMVECs). Cells were incubated with normal (5.5 mM) or high (33 mM) D-glucose concentrations for 5 days and then with 10 *μ*g/ml LPS for 12 h. The activity of horseradish peroxidase (HRP) in the lower compartment of the Transwell was measured by enzymatic assay to evaluate the permeability of PMVECs. NG, normal glucose; HG, high glucose; NG + LPS, normal glucose + LPS; HG + LPS, high glucose + LPS. Data are expressed as mean ± standard deviation (SD; n=5). ^*^P<0.05, compared with the NG group; ^#^P<0.05, compared with the HG group; ^▴^P<0.05, compared with the NG + LPS group.

We used HRP as a marker for the endothelial cell barrier properties against macromolecules. Human PMVECs were cultured on polycarbonate Transwell membrane inserts. The PMVECs were incubated with normal (5.5 mM) or high (33 mM) D-glucose concentrations for 5 days in medium with 2% serum (to maintain the cells in a quiescent state) and then incubated with 10 *μ*g/ml LPS for 12 h. The amount of HRP passing from the upper to the lower compartment was measured by enzymatic assay. As shown in Fig. 5, compared with the HG control group, the amount of HRP that passed through the membrane was significantly increased in the HG + LPS group (both P<0.05). The amount of HRP that passed through the membrane was also significantly higher in the HG + LPS group than in the NG + LPS group, (P<0.05).

### Expression of DDAH-2, eNOS and iNOS in human PMVECs

**Figure 6.**
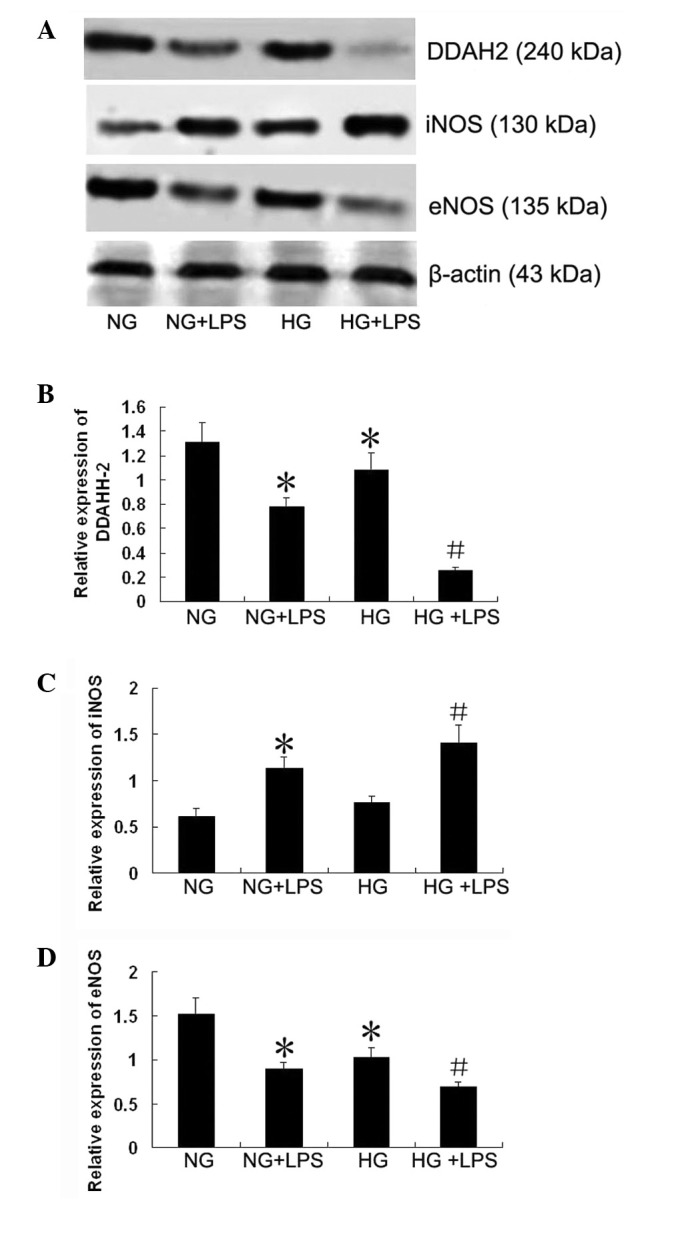
Effect of lipopolysaccharide (LPS) and a high glucose concentration on expression of dimethylarginine dimethylaminohydrolase (DDAH)-2, inducible nitric oxide synthase (iNOS) and endothelial NOS (eNOS) in cultured human pulmonary microvascular endothelial cells (PMVECs). Cells were incubated with normal (5.5 mM) or high (33 mM) D-glucose concentrations for 5 days in medium with 2% serum (to maintain the cells in the quiescent state), and then incubated with 10 *μ*g/ml LPS for 12 h. Cellular protein was isolated, separated by sodium dodecyl sulfate-polyacrylamide gel electrophoresis (SDS-PAGE) and transferred to membranes for western blotting as described in Materials and methods (A). Densitometry was performed to quantify the expression (B–D). NG, normal glucose group; NG + LPS, normal glucose + LPS group; HG, high glucose group; HG + LPS, high glucose + LPS group. Data are expressed as mean ± standard deviation (SD; n=5). ^*^P<0.05, compared with the NG group; ^#^P<0.05, compared with the NG + LPS group.

As shown in Fig. 6, compared with the NG group, the protein expression level of DDAH-2 was significantly downregulated in the HG + LPS group (P<0.05). The protein expression level of DDAH-2 was also significantly lower in the HG + LPS group than in the NG + LPS group (P<0.05). The expression level of iNOS in the NG + LPS group was significantly upregulated compared with that in the NG group (P<0.05). Compared with the NG + LPS group, the expression level of iNOS was significantly increased in the HG + LPS group (P<0.05). The expression level of eNOS in the HG + LPS group was significantly downregulated compared with that in the HG group (P<0.05). Compared with the NG + LPS group, the expression level of eNOS was significantly reduced in the HG + LPS group (P<0.05).

## Discussion

The pathway for water and small hydrophilic solutes through the walls of fenestrated vessels is through the fenestrae. Under specific conditions, the permeability of the microvasculature is increased such that macromolecules cross the endothelial barrier in three ways: through the intercellular junctions, through the endothelial cell fenestrae and transcellularly by shuttling vesicles and specific receptors (18). Observations made in large vessel endothelial cells have demonstrated that disrupting the actin cytoskeleton enhances vascular permeability (19). Fluorescent staining of human PMVECs revealed a marked effect on the organization of the F-actin filaments, the formation of stress fibers and intracellular gap formation in the presence of NG and LPS. However, HG concentrations and LPS strongly effaced actin at the cell periphery with thin or short cortical actin, induced the formation of abundant quanties of stress fibers and resulted in the appearance of small paracellular gaps. The damage to these actin and junctional protein bonds results in the dissociation and redistribution of proteins, which affects the cell-to-cell barrier functions. In the present study we observed a significant augmentation of the number and diameter of fenestrae of endothelial cells during a 24-h period of treatment with LPS. Moreover, a similar but significantly stronger increase in the number and diameter of fenestrae was observed following a 24-h HG and LPS treatment; this facilitated the passing of water through the cell monolayer and ultimately increased macromolecule permeability.

The results of the current study also demonstrated that the HG concentration and LPS induced an increase in the permeability of the cultured endothelial monolayer to macromolecules, including HRP, which is a marker for determining the endothelial barrier permeability. HRP flux across the endothelial cell monolayer was 2.3-fold higher in the HG + LPS group compared with the NG + LPS group. Evidently, there is a strong synergy between the effects of HG concentration and LPS on endothelial barrier permeability. These findings indicate that hyperglycemia associated with infection may create an early window of vulnerability allowing secondary insults to activate deleterious endothelial functions.

Injury to endothelial cells is a key mechanism for acute lung injury (ALI) and its most severe form, acute respiratory distress syndrome or sepsis, by which microvasculature permeability increases (20–22). Under such hyperglycemic circumstances, such damage may be more evident.

NO is synthesized from L-arginine by NOS. nNOS and eNOS are referred to as constitutive NOS (cNOS). NO production by cNOS modulates several aspects of intestinal physiology and is considered to be required for maintaining epithelial cell barrier integrity (23). In the present study, compared with a NG concentration, a HG concentration resulted in a reduction of LPS-stimulated eNOS expression. Reduced levels of NO formation by eNOS may be due to reductions in eNOS expression levels, deficiency of its cofactor, tetrahydrobiopterin (BH4) or eNOS translocation to a membrane compartment distant from the plasma or Golgi membranes (24). Moreover, eNOS activity is regulated by endogenous inhibitors, particularly ADMA, which is metabolized by DDAH. Therefore, DDAH is a determinant for ADMA concentrations and its dysregulation may have an important role in ADMA elevation and NOS pathway modulation in pathological conditions, including hyperglycemia. In the mouse lung, eNOS protein expression is reduced 12 h after LPS treatment (25).

By contrast, NO is produced by iNOS under pathological conditions, including inflammation, and it reduces the integrity of the intestinal epithelium. Several studies have shown that iNOS is induced by bacterial products, microbes and certain cytokines, resulting in the production of high NO levels. NO overproduction and the attendant oxidative injury to key proteins are necessary for dysregulation of the monolayer barrier and barrier hyperpermeability (26–28). Additional studies have identified that increased lung NO production is associated with increased iNOS expression and/or iNOS activity in various ALI models (29,30). In the current study, we demonstrated that LPS increased NO production and iNOS expression but reduced eNOS expression, suggesting that the increased NO levels may be iNOS derived.

Previous studies have demonstrated that the ADMA/ DDAH pathway regulates pulmonary endothelial barrier function by modulating Rac1 signaling (31). We demonstrated that hyperglycemia boosts LPS-initiated NO production by enhancing iNOS expression, as well as reducing DDAH-2 expression. However, enhanced ADMA levels are not sufficient to limit NO production by iNOS; however, it may contribute to reducing NO production by eNOS (17). iNOS may compete with eNOS by limiting BH4 availability. Furthermore, nitrosative stress induced by iNOS overexpression reduces DDAH activity.

In summary, in the current study we demonstrated that the exposure of human PMVECs to a HG concentration increased LPS-stimulated permeability. This may be linked to dysregulation of the NO pathway. Therefore, monitoring glucose control may be important for those in acute stress to protect endothelial cells from secondary damage processes.

## Figures and Tables

**Table I.** Number and size of fenestrae in human PMVECs.

Group	No. of fenestrae per *μ*m^2^	Average diameter of fenestrae (nm)
NG	4.87±0.74	8.29±1.76
HG	12.89±2.32^a^	17.60±5.59^a^
NG + LPS	24.0 0±2.19^a^	31.25±9.57^a^
HG + LPS	38.43±5.08^b^	52.07±16.40^b^

Pulmonary microvascular endothelial cells (PMVECs) were incubated with normal (5.5 mM) or high (33 mM) D-glucose concentrations for 5 days, then with 10 *μ*g/ml LPS for 12 h. The samples were prepared for scanning electron microscopy. NG, normal glucose; HG, high glucose; NG + LPS, normal glucose + LPS; HG + LPS, high glucose + LPS; LPS, lipopolysaccharide. Data are expressed as mean ± SD (n=5).

a P<0.05, compared with the NG group;

b P<0.05, compared with the HG or NG + HG group.
